# Elizabethkingia miricola: an opportunistic pathogen in ICU: Eva Soler

**DOI:** 10.1093/eurpub/ckac131.406

**Published:** 2022-10-25

**Authors:** E Soler, E Marín, I Guerrero, S Martínez, MA Fernández, C Valero

**Affiliations:** Medicina Preventiva y Salud Pública, Hospital Clínico San Cecilio, Granada, Spain

## Abstract

**Background:**

The Elizabethkingia genus is formed by a group of bacteria which are widely distributed in nature. Elizabethkingia is not part of human microbiota, therefore is considered to be an opportunistic pathogen. In the last years, it has become a cause of potentially fatal disease, becoming an emerging bacteria of increasing relevance. The objective of this study is to describe the impact of Elizabethkingia bacteria in infected patients in the ICU of a hospital in Granada.

**Methods:**

Descriptive study. Patients who have been isolated in a biological sample of Elizabethkingia miricola throughout the year 2.021 in the ICU of San Cecilio University Hospital in Granada. Date and place of isolation were registered. Other variables registered were sex, age, length of ICU stay, days between ICU admission and bacterium isolation, days between bacterium isolation and death, infection, cause of admission or cause of death.

**Results:**

Bacterium was isolated in 15 patients. Cause of admission was COVID-19 in 73.3% of patients. 73.3% were men and 26.6% women.The average age was 56.9 years. The average length of ICU stay was 43.8 days. 4 patients were diagnosed with ventilator-associated pneumonia and 5 patients were diagnosed with tracheobronchitis.The average days between ICU admission and bacterium isolation was 26,4 days. The average days between mechanical ventilation and bacterium isolation was 25.9 days. 53.3% of patients died. The average days between bacteria isolation and death was 18.2 days.

**Conclusions:**

Elizabethkingia miricola is an emerging bacterium under special vigilance due to its capacity to cause major morbidity and mortality in admitted patients in ICU. The rapid identification and the study of the antibiotic susceptibility is considered of special relevance so they can be correctly managed to avoid infections and complications resulting from this microorganism.

**Key messages:**

• Elizabethkingia is a special surveillance bacterie due to its morbidity and mortality effects.

• Elizabethkingia could be a severity indicator in admitted patients to the ICU.

